# Molecular Characterization of Human Lymph Node Stromal Cells During the Earliest Phases of Rheumatoid Arthritis

**DOI:** 10.3389/fimmu.2019.01863

**Published:** 2019-08-20

**Authors:** Emmanuel Karouzakis, Janine Hähnlein, Cristoforo Grasso, Johanna F. Semmelink, Paul P. Tak, Danielle M. Gerlag, Steffen Gay, Caroline Ospelt, Lisa G. M. van Baarsen

**Affiliations:** ^1^Department of Rheumatology, Center of Experimental Rheumatology, University Hospital of Zurich, Zurich, Switzerland; ^2^Department of Rheumatology and Clinical Immunology, Department of Experimental Immunology, Amsterdam UMC, Amsterdam Infection and Immunity Institute, University of Amsterdam, Amsterdam, Netherlands; ^3^Amsterdam Rheumatology & Immunology Center (ARC), Academic Medical Center, Amsterdam, Netherlands; ^4^Flagship Pioneering, Cambridge, MA, United States; ^5^Ghent University, Ghent, Belgium; ^6^Cambridge University, Cambridge, United Kingdom; ^7^RxCelerate, Cambridge, United Kingdom

**Keywords:** lymph node, stroma, fibroblast, rheumatoid arthritis, sequencing, DNA methylation

## Abstract

Rheumatoid arthritis (RA) is a progressive, destructive autoimmune arthritis. Break of tolerance and formation of autoantibodies occur years before arthritis. Adaptive immunity is initiated in lymphoid tissue where lymph node stromal cells (LNSCs) play a crucial role in shaping the immune response and maintaining peripheral tolerance. Here we performed the first epigenomic characterization of LNSCs during health and early RA, by analyzing their transcriptome and DNA methylome in LNSCs isolated from lymph node needle biopsies obtained from healthy controls (HC), autoantibody positive RA-risk individuals and patients with established RA. Of interest, LNSCs from RA-risk individuals and RA patients revealed a common significantly differential expressed gene signature compared with HC LNSCs. Pathway analysis of this common signature showed, among others, significant enrichment of pathways affecting the extracellular matrix (ECM), cholesterol biosynthesis and immune system. In a gel contraction assay LNSCs from RA-risk individuals and RA patients showed impaired collagen contraction compared to healthy LNSCs. In RA LNSCs a significant enrichment was observed for genes involved in cytokine signaling, hemostasis and packaging of telomere ends. In contrast, in RA-risk LNSCs pathways in cancer (cell cycle related genes) were differentially expressed compared with HC, which could be validated *in vitro* using a proliferation assay, which indicated a slower proliferation rate. DNA methylation analyses revealed common and specific differentially methylated CpG sites (DMS) in LNSC from RA patients and RA-risk individuals compared with HC. Intriguingly, shared DMS were all associated with antigen processing and presentation. This data point toward alterations in cytoskeleton and antigen-processing and presentation in LNSC from RA-risk individuals and RA patients. Further studies are required to investigate the consequence of this LNSC abnormality on LNSC-mediated immunomodulation.

## Introduction

Rheumatoid arthritis (RA) is a progressive, destructive autoimmune disease of which the etiology is only partly understood. RA-specific autoantibodies as rheumatoid factor (RF) and anti-citrullinated protein antibodies (ACPAs) (1) can be present years before the manifestation of clinical disease (2) and during this RA-risk phase (3) synovial inflammation seems absent (4, 5), suggesting that possibly yet unidentified immune processes taking place outside of the synovium might promote disease development.

Since (auto-) antibody production and peripheral tolerance are initiated and maintained within secondary lymphoid organs, the lymph nodes (LN) stand out as promising extra-synovial target for investigation. In line with findings in mice, showing LN activation before onset of arthritis (6), we recently reported altered frequencies of B cells, T cell subsets and innate lymphoid cell subsets in LN biopsies of RA-risk and early-stage RA patients compared to healthy controls (7, 8).

Studies in mice revealed that lymph node stromal cells (LNSCs) are crucial regulators of adaptive immunity and highlighted their various abilities to shape T and B cell responses (9, 10). They also orchestrate trafficking of immune cells within the LN (11, 12), control lymphocyte activation, differentiation and survival (9), but can also inhibit T cell proliferation (13, 14) and maintain peripheral tolerance (13, 15). LNSCs comprise the CD31-expressing lymphatic endothelial cells (LECs) and blood endothelial cells (BECs) and the CD31 negative fibroblastic reticular cells (FRCs), which express podoplanin. According to their localization in the LN, FRCs can be further separated into marginal reticular cells (MRCs) and T-zone reticular cells (TRCs). In addition double-negative cells (DNCs) neither expressing CD31 nor podoplanin were described. This group of LNSCs contains follicular dendritic cells (FDCs) and other, less well described LNSC subtypes.

Since LNSCs have mainly been studied in mice, we developed an experimental model to allow research on human LNSCs (16). We recently reported clear disturbances in the microenvironment early in development of systemic autoimmunity (14, 16). LNSCs cultured from autoantibody positive individuals at risk of developing RA (RA-risk group) and RA patients showed reduced induction of chemokines and their capacity to regulate T cell proliferation seemed diminished (14, 16). Accordingly, due to their impact on immunity and tolerance, we hypothesize that malfunctioning of LNSCs leads to a microenvironment where immune responses are not properly controlled leading to activation of (autoreactive) lymphocytes and production of autoantibodies. Here we studied human LNSCs on a detailed molecular level by analyzing their genome-wide transcriptome and methylome profile during health and the earliest phases of RA to pinpoint epigenetic changes ongoing in systemic autoimmunity and to potentially identify new and unanticipated therapeutic targets.

## Results

### Molecular Cell Subsets of Expanded Human LNSCs in Culture

**Figure 1:**
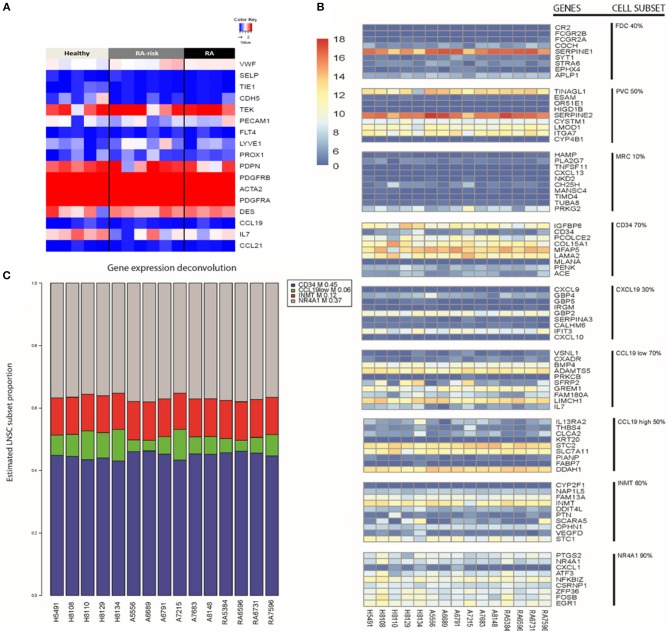
Key LNSC gene signature in human cells. **(A)** Heat map of expression data for genes characteristic for LNSCs as reported in mice is shown. Colors represent column-centered Z-scores of normalized read counts. Red is high, blue is low expression. **(B)** Different cell subsets present in bulk human LNSC cultures. The expression of the top 10 differentially expressed genes of each described murine LNSC subset (17) was investigated in the expanded human LNSCs. A heat map was obtained using the variance stabilizing transformation of the raw counts. The color scale indicates the low expressed genes (dark blue) up to the high expressed genes (dark red). Each column represents a donor, and each row refers to a gene. On the far right, the cell subset is given next to their related gene signature. The percentage indicates the proportion of genes in the subset signature expressed by our human LNSCs. **(C)** Propplot of the estimated cell subset distribution in cultured human LNSC. An NMF-class object that contains proportions as its mixture coefficient matrix (M). Each column refers to a donor. At the bottom of the column from left to right, healthy individuals (H), RA-risk individuals **(A)**, rheumatoid arthritis patients (RA). The colors correspond to the different cell subsets: CD34 (blue), CCL19low (green), INMT (red), NR4A1 (gray).

To study the molecular subtype of expanded human LNSCs, we first compared the measured expression profile with a transcriptional profile that was shown to differentiate FRCs, DNCs, LECs, and BECs in freshly isolated murine LNSCs (9). Based on gene expression of the human LNSCs, 12 out of 15 samples expressed podoplanin (PDPN), which differentiates FRCs (PDPN+) from DNCs (PDPN-) (Figure 1A).Flow cytometry analysis of CD31, CD45, and podoplanin (gp38) showed that the cultured human LNCS consisted of a mixed population of FRCs and DNCs with some variability in the proportion of each subtype, as also shown in our previous study (16) (Supplementary Figure 1).

Human LNSCs transcriptional profiles matched data from murine studies where FRCs and DNCs in contrast to BECs lack expression of von Willebrand factor (vWF), and selectin P (SELP), and in contrast to BECs and LECs lack expression of Tyrosine kinase with immunoglobulin-like and EGF-like domains 1 (TIE1), cadherin 5 (CDH5), CD31 (PECAM1), Fms-related tyrosine kinase 4 (FLT4), Lymphatic Vessel Endothelial Hyaluronan Receptor 1 (LYVE1), and Prospero homeobox protein 1 (PROX1) with clear expression of platelet-derived growth factor receptor (PDGFR) A and B, desmin and alpha-actin 2 (ACTA2). In contrast to the freshly isolated mouse LNSC, in the cultured human LNSC we found clear transcript levels of the Angiopoietin-1 receptor (TEK). Furthermore, none of the samples expressed CCL19 and CCL21 (Figure 1A), which might be due to the absence of external stimuli or contact with lymphocytes in *in vitro* cultured human LNSCs. Indeed, in an earlier study we showed that expanded human LNSCs are capable of producing these key chemokines upon stimulation, with lower induction observed in RA LNSCs (16). Overall, the transcriptional profile of *in vitro* expanded human LNSCs largely overlapped with the reported profile of mouse FRCs.

Recently, Rodda et al. looked into the cellular heterogeneity of murine peripheral LN (pLN) non-endothelial SCs using single-cell RNA sequencing (scRNAseq) (17). In this study, known cell types within the stromal cells family were identified, such as FDCs, MRCs, perivascular cells (PvCs), and TRCs. Importantly, this study identified 10 top-ranked genes differentially expressed between the above listed subtypes (Supplementary Table 1). From this analysis the TRC could be further discriminated into other subtypes called Ccl19high, Ccl18low, Cxcl9^+^, CD34^+^. The other two subtypes identified and named Inmt^+^ and Nra4^+^, could not be uniquely coupled to a described type of stromal cells (17).Further studies are needed to outline the function of these specific stromal cell subsets.

We investigated whether these subset specific gene signatures were expressed in our cultured human LNSCs (Figure 1B and Supplementary Table 2). The heatmap shows that many genes of the nine reported cell subsets are low expressed. As expected, our bulk LNSCs in culture do not contain FDCs, MRCs, and perivascular cells (PvCs), as these cells are lost during culturing. We next selected those cell subsets retaining more than 60% of the genes from the gene signature: CD34, INMT, CCL19low, and NR4A1 (Supplementary Table 2). Computational deconvolution (18) of global gene expression in our dataset suggests that our expanded LNSCs in culture mostly consists of the CD34, INMT, the CCL19low subsets, and NR4A1 cells (Figure 1C and Supplementary Figure 2). Looking at the expression of the gene set that was used by Malhotra et al. to define LNSC subsets, in the Rodda study showed that similar to the cultured human LNSCs, CD34, Inmt, Ccl19low, and Nr4a1 murine cells are characterized by low expression of Ccl19 and high expression of PDGFRA in contrast to the other SC subsets.

Healthy controls, RA-risk individuals and RA patients show no differential frequency of these cell subsets. This latter suggests that the original environment of LNSC (healthy, RA-risk or RA) do not determine the preferential outgrowth of one subset over another and that potential differences between donors are probably unrelated to differences in cell subsets present.

### Transcriptional Changes in RA-Risk and RA LNSCs When Compared to Healthy LNSCs

**Figure 2:**
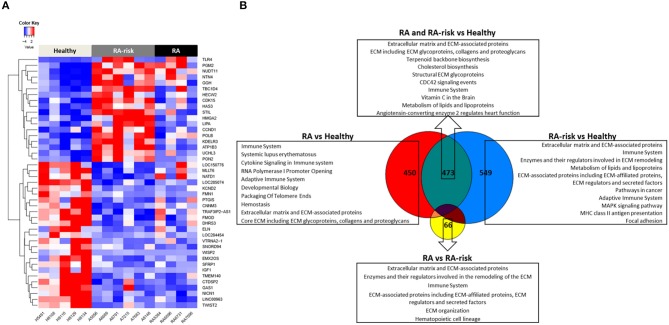
Transcriptomic analysis of human LNSCs. **(A)** Cluster diagram representing the most significantly differentially expressed genes in LNSC when comparing RA(-risk) with healthy controls using an FDR *q* < 0.05. Colors represent Z-scores of normalized read counts (row-scaling). Red is high, blue is low expression. **(B)** Venn diagram indicating significantly differentially expressed genes between the 3 study groups using a cutoff of *P* < 0.05, log2 fold change >0.5, and a mean expression >50. Subsequently, the Molecular Signature Database was used to compute the overlap between the obtained significantly differentially expressed gene lists and gene lists in well-described pathway databases (Canonical pathways, KEGG, Biocarta, and Reactome). The top 10 pathways are listed with an FDR *q* < 0.05.

Next we compared the transcriptional profiles of healthy (HC, *n* = 5), ACPA + RA-risk (*n* = 6), and RA (*n* = 4) LNSCs using RNA sequencing. Despite being limited by a small number of LNSC donors, this explorative analysis revealed genes that were significantly differentially expressed in LNSCs from RA/RA-risk individuals compared with HC. One-way hierarchical clustering based on the most significantly differentially expressed genes between the groups (FDR < 5%, fold change ± 0.5, and mean expression > 50) indicated that RA/RA-risk LNSCs displayed a distinct expression profile compared to healthy control LNSC, while LNSCs from RA-risk individuals and RA patients were more similar to each other (Figure 2A). This became particularly clear when analyzing the overlap in differentially expressed genes (based on *P* < 0.05, fold change ± 0.5, and mean expression > 50) between the three study group comparisons (Figure 2B). The Venn diagram revealed only minor differences between LNSCs from RA-risk patients when compared to LNSCs from RA patients (66 genes).

**Figure 3:**
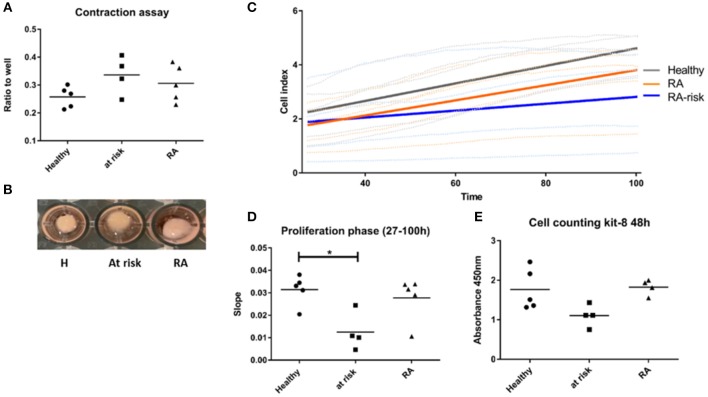
Functional assays supporting findings of transcriptional profiling. **(A)** Contraction assay. Cells were seeded in collagen gels and gels released after 48 h. Contraction of gel was measured after 20 h. **(B)** Representative picture of contraction assay. **(C)** Proliferation of LNSCs was analyzed using the xCelligence system which measures adhesion, spreading and proliferation in real time during culture. Picture shown is focusing on the proliferation phase starting 27 h after seeding (see also Supplementary Figure 3). RA-risk LNSCs proliferated slower compared to healthy controls. **(D)** Quantification of results shown in **(C)**. **(E)** Cell counting kit-8: Cells were seeded at equal numbers and the amount of formazan dye, generated by dehydrogenase activity in cells and being proportional to the amount of living cells, was measured after 48 h. *p < 0.05.

In contrast, quite a high number of genes were differentially expressed either specifically in RA (450) or RA-risk (549) LNSCs or in both (473) when compared to HC. Genes that were only significantly changed in healthy vs. RA or healthy vs. RA-risk, but were still not significantly changed in the RA/RA-risk comparison, usually showed a change in the same direction, but did not reach statistical significance according to our cut-offs (*p* < 0.05, fold change ± 0.5, and expression >50 in all measured samples). These numbers thus underline the similarity of RA-risk and RA LNSCs. Pathway analysis of the commonly differentially expressed genes in RA/RA-risk LNSCs showed, among others, significant enrichment of genes encoding for proteins or regulators involved in extracellular matrix (ECM) including many genes involved in the actin cytoskeleton (Figure 2B). To validate and interpret this finding a gel contraction assay was performed which revealed that LNSCs from RA-risk individuals and RA patients showed impaired collagen contraction compared to HC (Figures 3A,B). The gels containing healthy LNSCs (*n* = 5) covered 26.5% ±2.5 of the well, while RA-risk (*n* = 4) and RA (*n* = 5) LNSC only contracted the gels to 33.9% ±5.9 and 30.6% ±6.5, respectively (Figure 3B representative picture).

Other common significantly enriched genes were, amongst others, involved in cholesterol biosynthesis and the immune system.

Of the transcripts that were differentially expressed specifically in either RA-risk or RA LNSCs when compared to healthy LNSCs, gene-set analyses showed similar enrichment of pathways associated with ECM and the immune system. RA LNSCs specific enrichment involved pathways like Systemic lupus erythematosus (e.g., C3), cytokine signaling (e.g., IL-7) and packaging of telomere ends (e.g., histone cluster genes; Figure 2B). In contrast, specific gene-sets enriched in RA-risk LNSCs included pathways involved in cancer (e.g., cell cycle related genes), MHC class II antigen presentation and focal adhesion.

To confirm specific differences in RA-risk LNSCs on a functional level, we analyzed adhesion, spreading, and proliferation of LNSCs using the xCelligence system, which measures cell growth viability in real time during culture (19). When focusing on the proliferation phase, RA-risk LNSCs proliferated slower compared to healthy controls (Figures 3C,D and Supplementary Figure 3). This finding could be confirmed by determination of the cell numbers after 48 h of cell culture (Figure 3E).

Collectively, compared with healthy controls, LNSCs from RA-risk individuals and RA patients displayed a different transcriptional gene expression profile indicating altered proliferation and contraction of LNSCs in RA-risk and RA patients, respectively.

### Differences in DNA Methylation in RA (-Risk) LNSCs

**Figure 4:**
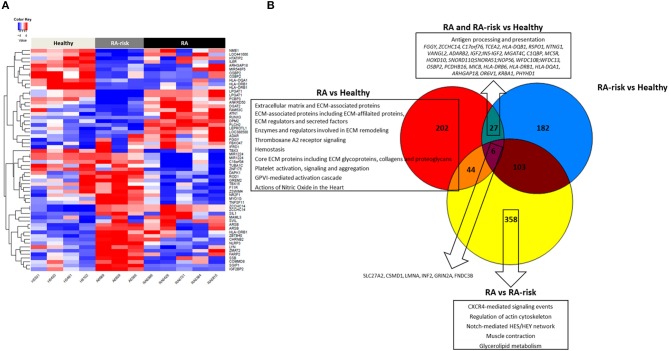
DNA methylome profiles of human LNSCs. **(A)** Cluster diagram representing the most significantly differentially expressed genes in LNSC when comparing RA(-risk) with healthy controls. Plotted are the beta values of the top 20 CpG gene sites (top 10 genes with mean average beta values above 0.3 are methylated in red and top 10 genes with mean average beta values of above −0.3 delta are unmethylated in blue) per comparison. **(B)** Venn diagram indicating significantly differentially methylated CpG sites between the 3 study groups using a cutoff mean beta value differences of 0.1 and *p*-values below 0.05. Subsequently, the Molecular Signature Database was used to compute the overlap between the obtained significantly differentially methylated CpG gene sites and gene lists in well-described pathway databases (Canonical pathways, KEGG, Biocarta, and Reactome). If present, the top 10 pathways are listed with an FDR *q* < 0.05.

DNA methylation analyses uncovered 279 significantly differentially methylated CpG sites (DMS) in LNSCs from RA patients (*n* = 5) vs. HC (*n* = 4), 318 DMS between RA-risk individuals (*n* = 3) vs. HC, and 511 DMS when comparing RA patients with RA-risk individuals (mean beta value differences of 0.1, *p* > 0.05). One-way hierarchical clustering of the top 20 CpG gene sites (top 10 with mean beta value difference of above 0.3 are defined methylated and top 10 with −0.3 are defined unmethylated) per comparison in a heatmap clearly shows different DMS patterns between healthy, RA-risk and RA LNSCs (Figure 4A).

Key immune related genes showing different methylation patterns included IL-6R and HLA-DR. Next, we again plotted the DMS into a Venn diagram to indicate common and specific DMS (Figure 4B). Six DMS (*SLC27A2, CSMD1, LMNA, INF2, GRIN2A, FNDC3B*) were identified that were commonly differential in RA/RA-risk vs. healthy LNSCs but also different between RA and RA-risk LNSCs. Pathway analysis on specific DMS in RA LNSCs showed again an enrichment of regions involved in ECM and hemostasis whereas in RA-risk LNSCs no significant enrichment could be detected. Twenty seven DMS were different in both RA and RA-risk LNSCs compared to healthy LNSC. Eighty percent of all DMS were significantly hypomethylated and pathway analyses showed that these DMS are mostly involved in antigen processing and presentation as they include HLA-DR gene regions.

**Figure 5:**
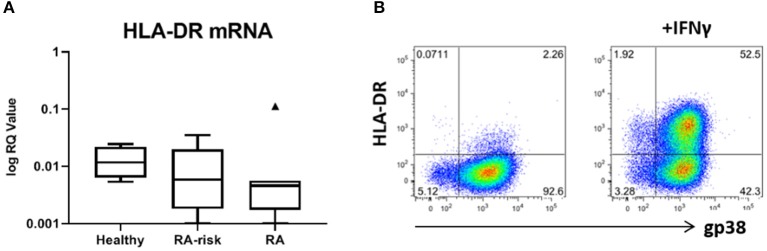
Expression of HLA-DR by human LNSCs. **(A)** mRNA expression levels of HLA-DRA was assessed by qPCR and data is presented in Tukey boxplots (healthy *n* = 7, RA-risk *n* = 8, RA *n* = 7). **(B)** Flow cytometry gating strategy used to identify CD45-CD31- stromal cells according to their podoplanin (gp38) and HLA-DR (MHC class II) expression. Gating was based on negative isotype staining. Pictures display 1 representative donor out of 13 donors tested (healthy *n* = 5, RA-risk *n* = 5, and RA *n* = 3). Numbers adjacent to the outlined areas indicate percentage of cells in the gated population.

We next investigated the expression levels of HLA-DRA mRNA in our cultured LNSCs. Under homeostatic conditions HLA-DRA was expressed at low levels, with a trend toward lower levels in RA LNSCs (Figure 5A). To check the surface expression of HLA-DR on our expanded LNSCs we stimulated our cells with IFNγ, which has been described to upregulate HLA-DR on LNSCs (20, 21). Flow cytometry revealed that only a minor frequency of unstimulated LNSCs expressed HLA-DR on their surface (22), while IFNγ stimulation rapidly induced HLA-DR (Figure 5B) with similar levels in RA/RA-risk LNSCs and healthy LNSCs (data not shown).

Overall, this data reveals epigenetic imprinting of LNSCs during the earliest phases of RA.

## Discussion

In this explorative study, we characterized for the first time the epigenomic landscape of human LNSCs during health and systemic autoimmunity. We demonstrate that cultured human LNSCs show a comparable expression profile to mouse LNSCs, suggesting that the reported functional role of mouse LNSCs is potentially also operational in humans. Furthermore, our findings clearly highlight that RA-risk and RA LNSCs are more comparable to each other than to healthy control LNSCs and that epigenetic changes already occur in autoantibody positive RA-risk individuals. Therefore, LNSCs seem to obtain an epigenetic imprint during systemic autoimmunity, which may play an important role in RA development.

Deconvolution analysis was performed to determine if the recently discovered murine LNSC subsets (17) could be present in our expanded human LNSCs based on bulk RNA sequencing data. The analysis shows that the expanded human LNSCs contains an heterogeneous population of LNSC subsets, where, based on the molecular profile, the so called INMT, CD34, and CCL19low are most predominant but also a low frequency of NR4A1 cells could be detected. The estimated frequency of the cell subsets was similar between LNSCs expanded from healthy controls and RA (-risk) individuals. This finding suggests that in our experimental setting the disease status does not promote the outgrowth of one subset of another one. However, we do not exclude that *in vivo* the disease status may affect the cell proliferation differently.

Our findings reveal disturbances within the LN microenvironment, which are in line with our earlier studies (8, 14, 16, 23, 24) and with reports showing defects of the stromal compartment within the bone marrow (BM), another very specialized niche next to the LN. For RA several defects in the BM in stromal but also in hematopoietic cells have been reported (25, 26). The frequency of polyreactive antibodies found in naïve B cells is higher in RA than in healthy controls suggesting a defect B cell central tolerance checkpoint within the BM (25). In addition, long-term cultures of mononuclear cells derived from BM aspirates from RA patients failed to support normal haematopoiesis (27) and RA BM mesenchymal stromal cells (BM MSCs) in culture produced less IL-7 (28). Our findings indicate a decreased proliferation of LNSCs in RA-risk compared with LNSCs from healthy controls. Myeloid progenitor cells in RA BM are also less proliferative compared to healthy controls (26). In addition in mouse models of systemic lupus erythematosus (SLE), BM MSC have a slower proliferation rate and differentiate less (29). Impaired proliferation of RA-risk LNSCs might influence their immune-modulatory activities.

Proliferation and contraction of LNSCs are important features of stromal cells in order to regulate LN flexibility and integrity during ongoing inflammation (30, 31). Expansion of LNs occurs during an immune response to accommodate the increasing number of lymphocytes, followed by contraction and return to steady state. The initial expansion of the LN is greatly regulated by the podoplanin-CLEC2 signaling axis. Podoplanin, which is expressed on two subsets of LNSCs, FRCs, and the LECs, binds to CLEC-2 on activated, infiltrating dendritic cells (DCs) which leads to release of the stiffness of the FRC network, allowing them to stretch and it also induces proliferation (32). In parallel with FRCs, also lymphoid tissue inducer cells (LTi's) proliferate during infection as seen in mouse models using Lymphocytic Choriomeningitis Virus (LCMV) (31). LTi's in turn increase their release of LTα_1_β_2_ needed for stromal cell maturation and stimulates production of IL-7 by LNSCs (10, 31, 33, 34), which drives LTi survival. Crosstalk between FRC and LTi's is essential to foster LN expansion, remodeling and restoration after infection (35). A previous study in our lab indicates that the number of LTis is decreased in LN biopsies of RA patients and RA-risk individuals (8). Altogether, the reduced capacities of RA-risk LNSCs to contract and proliferate and the reduced numbers of LTis might hamper the function of the LNSCs during immune responses. Accordingly, the LN microenvironment in RA-risk might be less capable to cope with inflammatory conditions. The more pronounced changes in contraction and proliferation in RA-risk individuals compared to RA patients might also reflect a normal activation reaction in at-risk LNSCs, which is impaired in RA LNSCs. Further investigations and functional analysis of LNSCs from acute immune activation and chronic autoimmune activation are needed to clarify these points.

DNA methylation analyses revealed several differentially methylated CpG sites (DMS) in LNSCs from RA and RA-risk compared to healthy LNSCs. Of interest, the most profound differences between RA/RA-risk and healthy LNSCs were found in hypomethylated DMS involved in antigen processing and presentation pathways (HLA-DR gene regions). Under resting conditions LNSCs have a higher expression of MHC class I and II as well as antigen-processing machinery than skin fibroblast (9), and LNSCs upregulate genes within the MHC class II pathway during an immune response (9), which taken together marks them as superior antigen presenters among fibroblasts. Hypomethylation of CpGs makes the chromatin more accessible and genes potentially easier and faster to transcribe. For instance, if murine skin epithelial stem cells were primed earlier *in vivo* by a tissue damage, epigenetic changes in the chromatin surrounding stress response genes occurred. As a result, these cells reacted more rapidly to the next challenge (36). It is tempting to speculate that LNSCs increase their antigen presentation during systemic autoimmunity, based on epigenetic changes introduced by a stimulus in the early, at-risk phase of the disease. However, since antigen presentation in LNSCs is mainly described to induce tolerance (34), further experiments are needed to investigate whether LNSCs try to limit or induce autoimmunity. Furthermore, we also observed clear differences in DNA methylation between RA-risk and RA LNSCs, suggesting ongoing epigenetic changes during disease progression. This is in line with data shown in synovial fibroblasts from RA patients, where methylation patterns only partially overlapped between patients with early RA and patients with long-term established RA (37, 38). Accordingly, longer follow up experiments should be performed to better delineate the role of epigenetic changes in LNSCs during RA progression.

In conclusion, our explorative study shows clear changes in the epigenetic and transcriptomic landscape of human LNSCs in RA-associated autoimmunity. However, only a small number of individuals have been analyzed and the variation between donors is high, therefore these findings should be evaluated in a larger cohort. Also further challenging mechanistic studies are required to investigate whether altered epigenetics in LNSCs from RA/RA-risk patients alter the function of these cells. Our approach of characterizing LNSC during RA development has great potential to reveal new and unanticipated pathogenic processes that contribute to RA and might discover promising new therapeutic targets.

## Materials and Methods

### Study Subjects and Lymph Node Biopsy Sampling

We included individuals with arthralgia and elevated IgM-RF and/or ACPA levels, but without any evidence of arthritis upon examination (RA-risk individuals, phase c/d; *n* = 8) (3). The Median follow up time of these RA-risk individuals was 20.4 months [9.8–35.6 (IQR)] and none of the RA-risk individuals developed arthritis during this period. RA-risk individuals were not allowed to have systemic or intra-articular corticosteroid injection <28 days before enrolment. In addition, RA patients with established disease based on fulfillment of the American College of Rheumatology and European League Against Rheumatism (ACR/EULAR) 2010 (39) criteria and as assessed by the rheumatologist were included (*n* = 8). Healthy individuals without any joint complaints and without elevated IgM-RF and/or ACPA level and without active viral infection or any history of autoimmunity or malignancy and no present or previous use of disease-modifying antirheumatic drugs (DMARDs), biologicals or other experimental drugs served as control group (*n* = 8). IgM-RF was measured using IgM-RF ELISA {Hycor Biomedical, Indianapolis, IN [ULN (upper limit of normal) 49 kU/mL]} and ACPA was measured using anti-CCP2 ELISA CCPlus [Eurodiagnostica, Nijmegen, the Netherlands (ULN 25 kAU/L)]. The study was performed according to the principles of the Declaration of Helsinki, approved by the institutional medical ethical review board of the Academic Medical Centre, and all study subjects gave written informed consent. All study subjects underwent an ultrasound-guided inguinal LN needle core biopsy as previously described (40). At the day of LN sampling none of the donors showed signs of an infection. Table 1 shows the demographics of the included subjects.

**Table 1:** Demographic data of study subjects.

	**Healthy controls** ***n* = 8**	**RA-risk individuals** ***n* = 8**	**RA patient** ***n* = 8**
Sex (female) (*n*) (%)	6 (75)	8 (100)	5 (63)
Age (years) [median (IQR)]	24 (23–32)^*^	47 (36–56)	43 (34–59)
IgM-RF positive (*n*) (%)	0 (0)	0 (0)	5 (63)
IgM-RF level (kU/l) [median (IQR)]	—	3 (1–8)	34 (10–439)
ACPA positive (*n*) (%)	0 (0)	8 (100)	7 (88)
ACPA level (kAU/l) [median (IQR)]	—	130 (44–274)	614 (72–1765)
IgM-RF and ACPA both positive (*n*) (%)	0 (0)	0 (0)	5 (63)
DAS28 [median (IQR)]	—	—	4.4 (3.3–6.2)^a^
ESR (mm/h) [median (IQR)]	—	5 (3–9)	16 (4–32)^b^
CRP (mg/l) [median (IQR)]	0.4 (0.3–0.9)^a^	1.3 (0.7–2.9)	14.7 (1.7–70.3)^b^
68TJC [median (IQR)]	0 (0)	1.5 (0.3–3)	8 (3–16.5)^c^
68SJC [median (IQR)]	0 (0)	0 (0)	2 (1–11)^c^
Treatment (*n*) (%)			5 (63)
Corticoids			4 (50)
NSAID			2 (25)^d^
DMARD			3 (38)
Failed TNF inhibitor therapy			3 (38)

IgM-RF, IgM rheumatoid factor; ACPA, anti-citrullinated protein antibodies; ESR, erythrocyte sedimentation rate; CRP, C-reactive protein; TJC, tender joint count; NSAID, non-steroidal anti-inflammatory drug; DMARD, disease-modifying antirheumatic drugs.

a Levels missing from 2 individual.

b Levels missing from 4 individuals.

c Levels missing from 3 individuals.

d Levels missing from 3 individuals.

* Healthy controls are significantly younger than RA patients (P < 0.050, tested by Kruskal–Wallis followed by a post Dunn's test).

### Lymph Node Stromal Cell Culture and Stimulation

LNSC culture was performed as previously described (14, 16). In short, using a 70 μm cell strainer (BD Falcon, San Jose, CA) lymphocytes were depleted and the remaining stromal tissue of a freshly collected LN needle core biopsy was plated on a 6-well culture dish (Greiner CELLSTAR®, Sigma Aldrich, Zwijndrecht, the Netherlands) (passage 0; P0) and complete cell culture medium containing Dulbecco's Modified Eagle Medium (DMEM) low glucose (Gibco, Bleiswijk, the Netherlands) supplemented with 0.1% penicillin (Astellas Pharma Inc., Leiden, the Netherlands), 0.1% streptomycin, 0.05 mg/mL gentamicin, 10 mM HEPES buffer, 2 mM L-glutamine (all Gibco), and 10% fetal calf serum (FCS) (GE Healthcare, Zeist, the Netherlands) was added. Human LNSCs formed monolayers and were expanded by passaging using trypsinization [0.05% trypsin, 5 mM EDTA (Gibco)] in phosphate buffer saline (PBS, Fresenius Kabi,‘s Hertogenbosch, the Netherlands) for 7 min at 37°C. For harvesting, cells were washed with sterile PBS, trypsinized, and the cell suspension was collected and centrifuged for 10 min, 1,000 rpm (212 g) at 4°C. Cells were then resuspended in cold complete medium and counted using trypan blue (Sigma Aldrich) in a Bürker-Türk chamber (LO Labor Optik, Lancing, UK). As described previously, this *ex vivo* LNSC culture model contains a mixture of FRCs and DNs (16). For the experiments, cells were used between passages 4–8.

### RNA Sequencing Analysis

RNA was isolated with the RNeasy kit (Qiagen) and used for the generation of RNA libraries for sequencing with the Illumina TruSeq® Stranded total RNA preparation kit including eukaryotic ribo-depletion by microsynth AG (Balgach, CH). The libraries were sequenced with the Illumina NextSeq500 high output (1 × 75v2 instrument; >20 million reads per sample). Data reads were quality-checked with FastQC. Bioinformatic analysis included demultiplexing and trimming of Illumina adaptor residuals, mapping of reads to hg38 reference genome using STAR (2.5.1b), counting of mapped reads using HTSeq (0.6.0), and statistical analysis using DESeq2 (1.6.3). Transcripts with a *p*-value < 0.05, fold change ± 0.5, and expression >50 in all measured samples were considered as significantly changed between the groups (Supplementary Table 3). Deconvolution of the RNA-sequencing was performed in R (3.5.2) with CellMix (1.6.2) package using the Frobenius semi-supervised learning method (18). The gene signatures for each cell-type was retrieved from a publicly available dataset (17). Rodda et al. listed 90 murine genes which represent the expression of the top 10 DEG for all nine LN non-endothelial stromal cell clusters conserved across samples (Supplementary Table 1). The conversion from mouse to human nomenclature was done using biomaRt (version Ensembl Genes 95 for human, Mouse strains 95 for mouse). After the conversion, the gene selected for the stromal cell subset present in our dataset had to have a logCPM >2.5 in at least 12 samples as cut-off (Supplementary Table 2). Subsequently, we calculated the percentage of expressed gene signature present per stromal cell subset. Cell type was selected for deconvolution analysis if >60% of the murine genes of each list would be present in our dataset (Supplementary Table 2).

### Real-Time Cell Analyzer (RTCA)

Adhesion, spreading and proliferation was monitored with the xCELLigence Real-Time Cell Analyzer DP system (Omni Life Science). Cultured cells were detached with accutase (Sigma) and seeded in 16 well E-Plates (Omni Life Science) in quadruplicates (2,500 cells/well). The rate of proliferation was determined by calculating the slope of the cell index (CI) curve in the interval between 27 and 100 h after seeding.

### Cell Proliferation Assay

Cells were detached with accutase and seeded in 96 well plates (6000/well) in triplicates. After 48 and 96 h, respectively, Cell counting kit-8 solution (Dojindo) was added and incubated for 3 h before measuring the absorbance at 450 nm using a microplate reader.

### Contraction Assay

Cell contraction was measured in triplicates with a collagen-based contraction assay (Cell Biolabs) according to the manufacturers' instructions. Briefly, 50,000 cells were seeded in 200 ul collagen mix and medium was added after 1 h. After 48 h, gels were detached with a pipette tip. Pictures were taken after 20 h and the outline of the gel was measured with the AxioVision Software (Zeiss). The ratio of the gel outline over the well outline was taken as measure for the strength of the contraction.

### Flow Cytometry Analysis

Human LNSCs were harvested from a 6-well dish using 1 ml TripLE™ Select (Gibco) for 10 min at 37°C. Subsequently, cells were washed in PBA buffer (PBS containing 0.01% NaN3 and 0.5% bovine serum albumin (BSA) (Sigma Aldrich), and stained for 1 h with rat IgG2a anti-human Podoplanin (clone NZ-1, AngioBio, Huissen, the Netherlands) on ice. Afterwards cells were washed again in PBS buffer, followed by a second incubation for 30 min on ice protected from light using the following directly labeled antibodies: Polyclonal goat anti-rat IgG AlexaFluor647 (Invitrogen), CD45 FITC (clone HI30, Becton Dickinson (BD) Pharmingen, Vianen, the Netherlands), HLA-DR PE-Cy7 (clone L243, Sony Biotechnology, Surrey, UK) or with corresponding isotype control antibodies. Staining with HLA -ABC Pe-Cy7 (clone G46-2.6, Biolegend, London, UK) served as a positive control and was used to set-up the correct compensation configuration settings. Cells were measured on a FACS CANTO II (BD) and data were analyzed using FlowJo software 9.9.3 (Tree Star, Ashland, OR).

### Illumina Methylation Arrays

DNA was isolated from cultured fibroblasts with the DNA blood kit (Qiagen). DNA was converted by sodium bisulfite modification and hybridized to Infinium Human methylation 450k arrays. The raw intensity data (IDAT) were imported into R version 3.4.0 and processed using the minfi (1.22.1) Bioconductor package. All the samples passed the mean detection *p*-value of the minfi sample quality standard. Probes from the X and Y chromosome were removed to exclude sex variability. Individual beta-values for each sample were calculated for further statistical analysis. Beta values are defined as the ratio of methylated probe intensity and overall probe intensity (sum of both unmethylated and methylated probe intensity) (38).

### Differential Methylation Analysis

The COHCAP algorithm was applied for the statistical analysis of differentially methylated CpG sites and islands (41). A CpG site was counted as methylated if the beta-value was >0.4 and unmethylated if it was below 0.3. Differential methylated CpG sites (DMS) in promoter regions (DMP) and/or CpG islands that showed mean beta value differences of 0.1 and *p*-values below 0.05 were defined as significantly differentially methylated between the different conditions. Integrative Genome Viewer (IGV) was used to visualize the DMS. Heatmaps were computed using the entire set of significantly differentially methylated CpGs between different conditions.

### Pathway Analysis

Pathway analysis was performed using the “Investigate gene sets” option in the Molecular Signature Database (MsigDB) of the Broad Institute v6.1 (http://software.broadinstitute.org/gsea/msigdb/annotate.jsp). Differentially expressed genes were uploaded to compute significantly (FDR < 5%) enriched overlaps with the canonical pathway (CP, KEGG, BioCarta, and Reactome) collection in MsigDB.

### Quantitative Real-Time PCR

Total RNA was isolated using the RNeasy Mini kit or RNeasy Micro kit (Qiagen, Venlo, the Netherlands) according to the manufacturer's instructions, including a DNAse step to remove genomic DNA. Subsequently cDNA was prepared using the RevertAid H Minus First Strand cDNA Synthesis kit (Thermo Fisher Scientific, Landsmeer, the Netherlands). Quantitative PCR was performed using Taqman® Universal PCR master mix combined with Taqman assays (all from Applied Biosystems, Life Technologies, Zwijndrecht, the Netherlands). For detection we used a StepOnePlus™ Real-Time PCR System (Applied Biosystems). Values for HLA-DRA (Hs00219575_m1) were corrected by the expression level of 18S RNA (Hs99999901_s1). An arbitrary calibrator sample was used for normalization. For calculating the relative quantity (RQ) the delta-delta Ct method was used.

## Data Availability

The datasets generated for this study can be found in Array express E-MTAB-7897, E-MTAB-8085.

## Ethics Statement

The medical ethical committee of the Academic Medical Center Amsterdam approved this study and all study subjects gave written informed consent.

## Author Contributions

All authors were involved in drafting the manuscript or revising critically for important intellectual content and all the authors gave their approval of the final version of the manuscript to be published. DG, PT, CO, SG, and LvB: experimental design. EK, CO, CG, SG, JH, JS, and LvB: acquisition of data. EK, CO, CG, SG, JH, and LvB: analysis and interpretation of data.

### Conflict of Interest Statement

PT and DG are currently employed by Flagship Pioneering and RxCelerate, respectively. Both companies were not involved in the current study. The remaining authors declare that the research was conducted in the absence of any commercial or financial relationships that could be construed as a potential conflict of interest.
