# Supplementary material for: Molecular Characterization of Human Lymph Node Stromal Cells During the Earliest Phases of Rheumatoid Arthritis
Source: Front Immunol. 2019 Aug 20;10:1863. doi: 10.3389/fimmu.2019.01863 (PMC6711342; doi:10.3389/fimmu.2019.01863)
Supplement: Supplementary file 4 [file Data_Sheet_1.pdf]

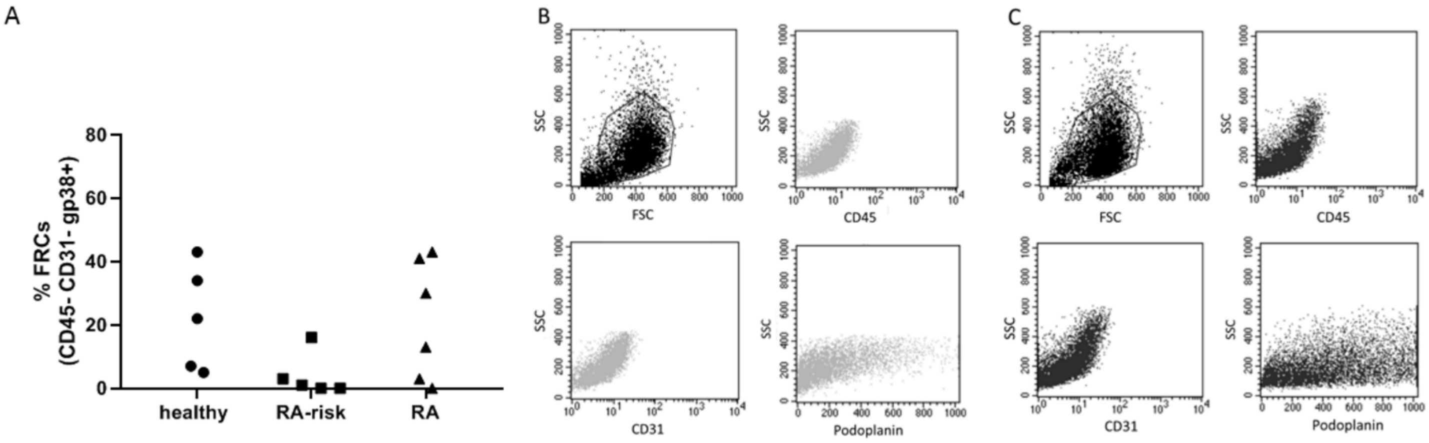

Supplementary Figure 1. Flow cytometry analysis of cultured LNCS.

A) Cultured LNSCs were a mixed population of fibroblastic reticular cells (FRCs; CD45- CD31- gp38+) and double negative cells (DNCs; CD45- CD31- gp38-). Representative figures of expression of CD45, CD31 and Podoplanin (gp38) in cultured LNCS from a samples with low (**B**) and high (**C**) expression of Podoplanin (gp38). FSC forward scatter, SSC side scatter
