# Supplementary material for: Molecular Characterization of Human Lymph Node Stromal Cells During the Earliest Phases of Rheumatoid Arthritis
Source: Front Immunol. 2019 Aug 20;10:1863. doi: 10.3389/fimmu.2019.01863 (PMC6711342; doi:10.3389/fimmu.2019.01863)
Supplement: Supplementary file 5 [file Data_Sheet_2.PDF]

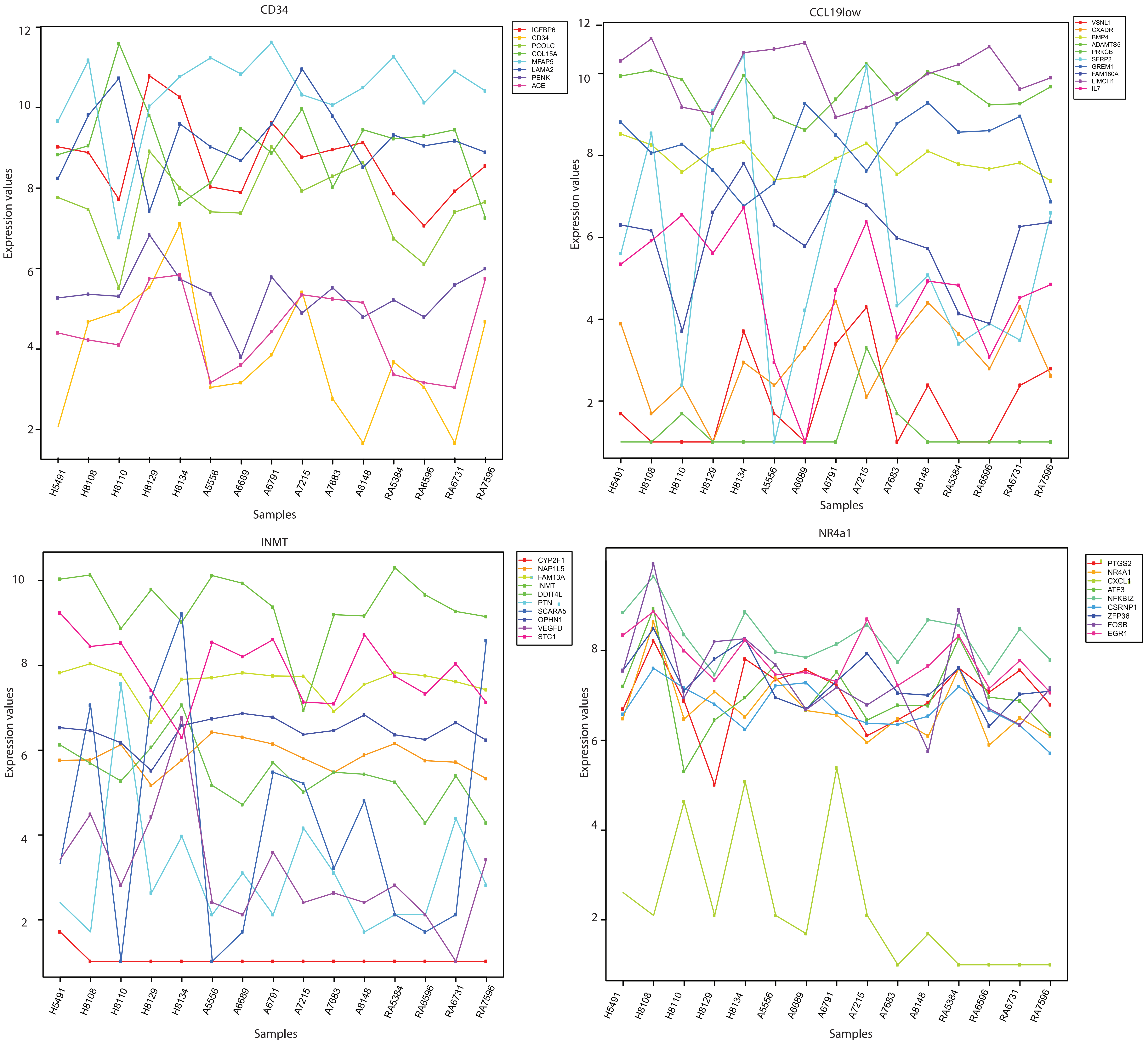

Supplementary figure 2. Contribution and distribution of genes per cell subset

From the top to the bottom for three cell subsets (CD34,CCL19low, INMT and NR4A1) expression values are given for each gene contributing to the specific gene signature . The y-axis indicates the expression values (logCPM) of each single gene in every sample (x-axis). On the top right corner of each graph, the color coded legend indicates the specific genes shown.
