# Supplementary material for: Molecular Characterization of Human Lymph Node Stromal Cells During the Earliest Phases of Rheumatoid Arthritis
Source: Front Immunol. 2019 Aug 20;10:1863. doi: 10.3389/fimmu.2019.01863 (PMC6711342; doi:10.3389/fimmu.2019.01863)
Supplement: Supplementary file 6 [file Data_Sheet_3.PDF]

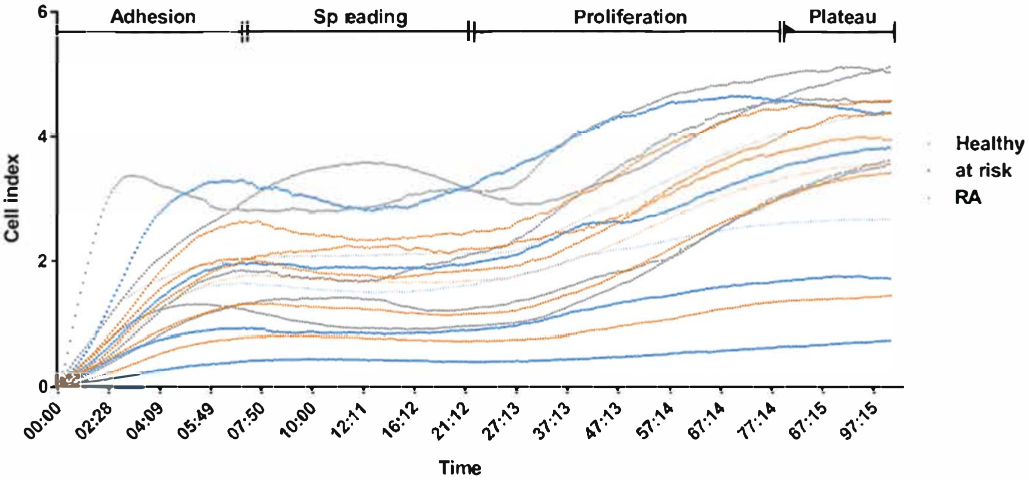

Supplementary Figure 3. Real-time proliferation assay Xcelligence
Proliferation of LNSCs was analyzed using the xCelligence system which measures adhesion, spreading and proliferation in real time during culture.
